# Influence of memorability on revisit intention in welcome back tourism: The mediating role of nostalgia and destination attachment

**DOI:** 10.3389/fpsyg.2022.1020467

**Published:** 2022-09-30

**Authors:** Yan Lu, Ivan Ka Wai Lai, Xin Yu Liu, Xin Wang

**Affiliations:** ^1^Institute for Research on Portuguese-Speaking Countries (IROPC), City University of Macau, Macao, Macao SAR, China; ^2^Faculty of International Tourism and Management, City University of Macau, Macao, Macao SAR, China

**Keywords:** welcome back tourism, memorability of a previous travel experience, nostalgia, destination attachment, revisit intention

## Abstract

“Welcome Back Tourism” is an important marketing strategy to help overseas Tourism destinations quickly recover from the crisis and enhance their core competitiveness. How to translate the memorability of tourists to revisit intention is the core key to open “Welcome Back Tourism.” This study takes local residents in Guangzhou, Shenzhen and Foshan as the research objects, and tries to explore the influence relationship between memorability of a previous travel experience, nostalgia, destination attachment and revisit intention. The results of 291 valid data showed that memorability of a previous travel experience had positive influence on revisit intention; Nostalgia has a positive effect on destination attachment. Nostalgia and destination attachment play a mediating role in the influence of memorability of a previous travel experience on revisit intention. The contributions and management Recommendations of these findings are discussed.

## Introduction

The phenomenon of tourist revisiting has received continuous attention from the tourism industry and academia because it can bring long-term sustainable development benefits to destinations (Zhang et al., 2021). Chinese tourists are the largest outbound tourism market group. Under the influence of culture and society, Chinese tourists tend to pursue novel experiences during overseas travel rather than pure leisure and relaxation (Cui et al., 2020; Chen et al., 2021). Therefore, Chinese tourists seldom visit the same destination. However, during the COVID-19 pandemic, Chinese tourists pay more attention to the recovery travel experience (Wen et al., 2020; Jin et al., 2021) and tend to choose destinations where they are familiar (Sun et al., 2022). Although at this moment, they return to the same domestic destinations, they will also return to foreign destinations once travel restrictions are lifted. Therefore, it creates an opportunity for overseas tourist destinations to attract returning Chinese tourists after COVID-19. But what drives their intention to revisit?

Welcome back tourism is a strategy widely used by tourism marketers to attract repeat visitors, which plays a crucial role in enhancing the core competitiveness of destinations (Eran and Eli, 2021). Providing unforgettable and valuable travel experiences for tourists is the key for a destination to win loyal customers (Kim, 2010). Therefore, the reason for tourists returning to a destination may be due to their memorable travel experiences. However, the memorable travel experience can be short-term memory and long-term. Most current research on the memorable travel experience is measured in situ, so it is based on tourists' short-term memory (Hu and Xu, 2021). People's memory of travel experiences will change over time. Tourists rarely revisit the same destination within a short period of time. Therefore, only the travel experience that has been stored and recalled for a long time can be regarded as valuable for welcome back tourism (Marschall, 2012). For evaluating the impact of long-term memory of tourist experiences, it needs to measure the memorability of previous travel experiences (MPTE) over time.

Nostalgia is a personal recollection of a good past, which stimulates people's positive emotions to cope with the unrealized present (Huang et al., 2016). At present, most studies have validated the cognitive assessment of nostalgia on tourist destinations and tourists, and few have focused on the impact of nostalgia on emotional or behavioral intentions, but its effect on revisit intention should exist. On the other hand, tourists are more likely to return to a particular destination if they have positive emotional connection and belief about it (Yuksel et al., 2010). It is a destination attachment. Previous studies have shown that destination attachment has a positive influence on revisit intentions (Cho, 2021; Jian et al., 2021). Nostalgia and destination attachment are both affective attitudes toward a tourist destination. These two attitudes can be evoked from MPTE according to Cognitive Assessment Theory (CAT). CAT stated that memory and emotion are interrelated. Once the memory network is activated, the arousal effect is activated. That is, people can evoke positive emotions such as personal nostalgia and attachment from memorability, and such positive emotions would affect tourists' intention to revisit (Kim et al., 2022). Therefore, this study suggests that over time, a highly memorable travel experience would trigger nostalgia and strengthen the emotional bond between tourists and destinations, and further encourage re-travel.

The purpose of this study is to explore how the MPTE affects the intention of Chinese tourists to revisit a destination under the mediating effects of nostalgia and destination attachment. There are three main contributions of this study. First, since previous studies on memorable travel experiences focused on short-term memory, this study focuses on long-term memory and makes a contribution to the research on memorable travel experience. Second, this study enriches welcome back tourism research by revealing the mechanism of how past travel experiences translate into revisit intentions. Third, some researchers in tourism started investigating the influence on past nostalgia on tourists' behaviors such as Zhang et al.'s (2021) study of tourists' autobiographical memory. This study contributes to tourism literature in providing researchers with more insights about nostalgia and its psychological responses of tourists. At the same time, this study has made significant practical implications for the marketing strategies of tourist destinations after the COVID-19 pandemic.

## Literature review

### Welcome back tourism

“Welcome back tourism” is a strategy to attract repeat visitors when a destination is in the recovery stage of crisis or disaster (Chacko and Marcell, 2008). The destination aims to persuade potential tourists to return and revisit the destination by evoking good memories of their previous stay at the destination and conveying the message “welcome back” to tourists. Studies have shown that repeat visitors are more likely to respond to reopening marketing messages and are most likely to be among the first to return after a crisis at a destination than those who have not visited (Mair et al., 2016). Therefore, the welcome back tourism strategy plays a crucial role in guiding destination managers to attract repeat visitors for recovery management.

Although the COVID-19 pandemic is definitely the catalyst for the welcome back tourism strategy trend, the phenomenon of tourist re-visit has been receiving continuous attention from the tourism industry and academia, as how to more effectively attract loyal tourists who repeat visits is always an important issue affecting the long-term sustainable development of destinations (Oppermann, 2000; Darnell and Johnson, 2001). Most studies have shown that attracting repeat visitors rather than new visitors is more beneficial to the long-term revenue of a tourism destination (Zhang et al., 2021). Since attracting repeat visitors is more beneficial to tourism destination promotion, researchers have paid much attention to the nature of the tourist revisit phenomenon, the reasons that influence the intention to revisit, and the factors that motivate tourists to revisit. Um et al. (2006) proposed that tourists' intention to revisit is largely motivated by their positive autobiographical memories. As a result, past memories are thought to be a key factor that may guide visitors' decision to revisit (Braunlatour et al., 2006).

### Memorability of a previous travel experience

The experience economy is centered on consumer experiences (Pine et al., 1999), and that experiences are valuable only if they can be translated into unique feelings and long-term memories (Clawson and Knetsch, 1966; Marschall, 2012). Tourism experience is the subjective assessment and psychological perception of tourists in the process of tourism (Otto and Ritchie, 1996). However, memorability refers to the ability of tourists to recall a specific event (Oh et al., 2007), and tourists' tourism recall is the result of the selective reconstruction of tourism experiences (Kim and Ritchie, 2014). In other words, not all tourism experiences can be judged as memorable by tourists. Only when tourists are actively remembered and recalled after experiencing tourism activities or events can they be called memorable travel experiences (Kim et al., 2012). Thus, memorability is the key result of tourism experiences (Chhetri et al., 2004).

Working memory, short-term memory, and long-term memory are all components of the complex cognitive system known as memory (Kim and Chen, 2021). At present, most studies on the memory of tourism experience are measured when tourists have just finished their trip (Sthapit et al., 2020). At the same time, there are few empirical studies on long-term memory, and the data obtained only reflect the short-term memory or working memory in which people temporarily store knowledge in their thoughts. According to the research of Craik and Lockhart (1972), the duration of memory storage depends on the level of memory entry processing. Meaningful stimuli can be stored in deep memory, and deep processing memory can be stored for a longer time, making it consolidated in the individual's mind for later retrieval. Marschall's (2012) study emphasizes the impact of people's long-term memory on destination choice by pointing out that people frequently return to locations connected to pleasant recollections of prior travels.

Some empirical studies have shown that there is a positive correlation between memory and repetition (Hung et al., 2016). For example, if the hotel can provide a memorable experience for customers, the return rate of customers may be increased (Ismail, 2010). Reliving great memories has been linked to memorable experiences, according to research by Tung and Ritchie (2011). When tourists store their previous travel experiences in a specific destination as good memories, they will show a stronger willingness to revisit and recommend it (Ali et al., 2016). If tourists have a long-term memory of a destination, they are willing to visit that destination again once the memory is evoked, even if the past trip was a long time ago. Therefore, the study hypothesizes the following:

**Hypothesis 1**: MPTE positively affects revisit intention.

### Nostalgia

The cognitive process of remembering the past and feeling its emotional effects is referred to as nostalgia (Chark, 2021). In psychological literature, nostalgia is often regarded as a “bittersweet” complex structure (e.g., Davis, 1979). Between the 17th and 19th centuries, nostalgia was understood as a “depressive” disorder of the mental and psychological systems (McCann, 1941). Affected by this negative reputation, nostalgia is understood as a negative emotion of sadness and loss such as endless longing for the past and mourning for the loss of time (Davis, 1979; Sedikides and Wildschut, 2016). However, current nostalgia is no longer regarded as a mental illness. Most researchers have proved that nostalgia is associated with a positive attitude (Batcho, 2013) and is an ideal recall emotion (Belk, 1990). And is described as a personal experience evoked by objects such as venue, atmosphere, music, and smell (Fairley and Gammon, 2005). Through the positive retelling of the past, people can feel positive emotions such as gratitude, joy, comfort, innocence and warmth (Jarratt and Gammon, 2016) and idealize the past (Pascal et al., 2002).

Nostalgia stems from personal memories of past experiences (Sedikides et al., 2015), and when the memories are positive or have higher value, individuals may be more inclined to repeat these memories, which leads to nostalgia (Marschall, 2014). The research of Chen and Chen (2010) showed that the unforgettable experience experienced by tourists in the consumption process will not end with the end of the consumption link, and the tourist experience will continue as personal nostalgia and interpersonal communication. Therefore, tourists have a long-term memory of a destination, and they will repeat this memory from time to time, so the MPTE leads to nostalgia. In this regard, this study puts forward the following hypothesis:

**Hypothesis 2**: MPTE positively affects nostalgia.

In general, nostalgia usually brings positive emotions such as warmth and happiness to the recalling person (Holbrook and Schindler, 1994; Kim and Moon, 2009), and these positive emotional reactions will lead to the generation of positive behavioral intentions. Studies have found that the feeling of nostalgia can help guide individuals' future attitudes and predict behaviors (Yeh et al., 2012). The study of Barnes et al. (2016) further pointed out that when tourists feel nostalgic for the travel experience, they often hope to recapture the experience by seeking a sense of familiarity, so they will have a higher willingness to revisit. Recently, Fairley et al. (2018) also found that cycling fans' nostalgia for past experiences drives their decision to revisit French mountain courses in the future. Therefore, tourists have a feeling of nostalgia for a tourist destination, and they are willing to revisit the destination. Therefore, this investigation advances the following hypothesis:

**Hypothesis 3**: Nostalgia positively affects revisit intention.

### Destination attachment

Destination attachment is not only a means of self-expression (Gross and Brown, 2006) but is also regarded as a kind of relationship construction (Park et al., 2010), that is, the emotional bond formed between tourists and tourist destinations (Kumar, 2016). Destination attachment, in accordance with attachment theory, is a reaction to an experience related to a specific place, which makes tourists tend to trust the destination (Bowlby, 1980) and exerts an important influence on the attitude and behavior of tourists (Prayag and Ryan, 2012).

When tourists are deeply impressed or highly familiar with the travel experiences of a certain destination, that valuable and unforgettable experiences will stimulate their various emotions toward the destination, and the resulting strong feelings will make them more attached to the destination (Tsai, 2016; Sthapit et al., 2022b). Previous studies have confirmed that memory makes an important component of destination attachment (Sthapit et al., 2018, 2022a; Cifci, 2022). Therefore, tourists have a long-term memory of a destination, they will have a strong feeling toward the destination, and which makes them have an attachment to the destination. Therefore, the following hypothesis is put forward:

**Hypothesis 4**: MPTE positively affects destination attachment.

Destination attachment is related to arousing or stimulating meaningful emotions of individuals, and these positive emotions will affect their attitudes and behaviors (Yuksel et al., 2010). Previous studies have shown that destination attachment can predict revisit intention (Yuksel et al., 2010; Prayag and Ryan, 2012), and the degree of attachment determines the strength of behavioral intentions (Japutra et al., 2018; Jian et al., 2021). Based on the theory of planned behavior, tourists' attitudes toward a destination influence their travel behavioral intentions. When tourists feel attached to a certain destination, the intensity of the emotional relationship between the individual and the destination will enhance the intention to revisit, that is, the stronger the destination attachment, the higher the intention to revisit (Japutra and Keni, 2020; Cho, 2021; Jian et al., 2021). Based on this principle, the following hypothesis is put out:

**Hypothesis 5**: Destination attachment positively affects revisit intention.

Previous studies have shown that there is a strong connection between nostalgia and destination attachment (Dai, 2017), that is, the stronger the nostalgia of tourists, the deeper their attachment to the destination (Tsai et al., 2020; Cho, 2021). Nostalgia is the recollection of tourists' beautiful tourism experiences and arouses their desire to return to past unforgettable experiences (Sedikides et al., 2015). Since nostalgia is a kind of positive emotion generated by positive experiences in the past (Tsai et al., 2020), tourists' nostalgia will bring unique meaning to them the destination (Cho, 2021), thus, inducing them to form an attachment to the destination (Sthapit et al., 2022b). Based on this, the following hypothesis is proposed:

**Hypothesis 6**: Nostalgia has a positive effect on destination attachment.

**Figure 1:**
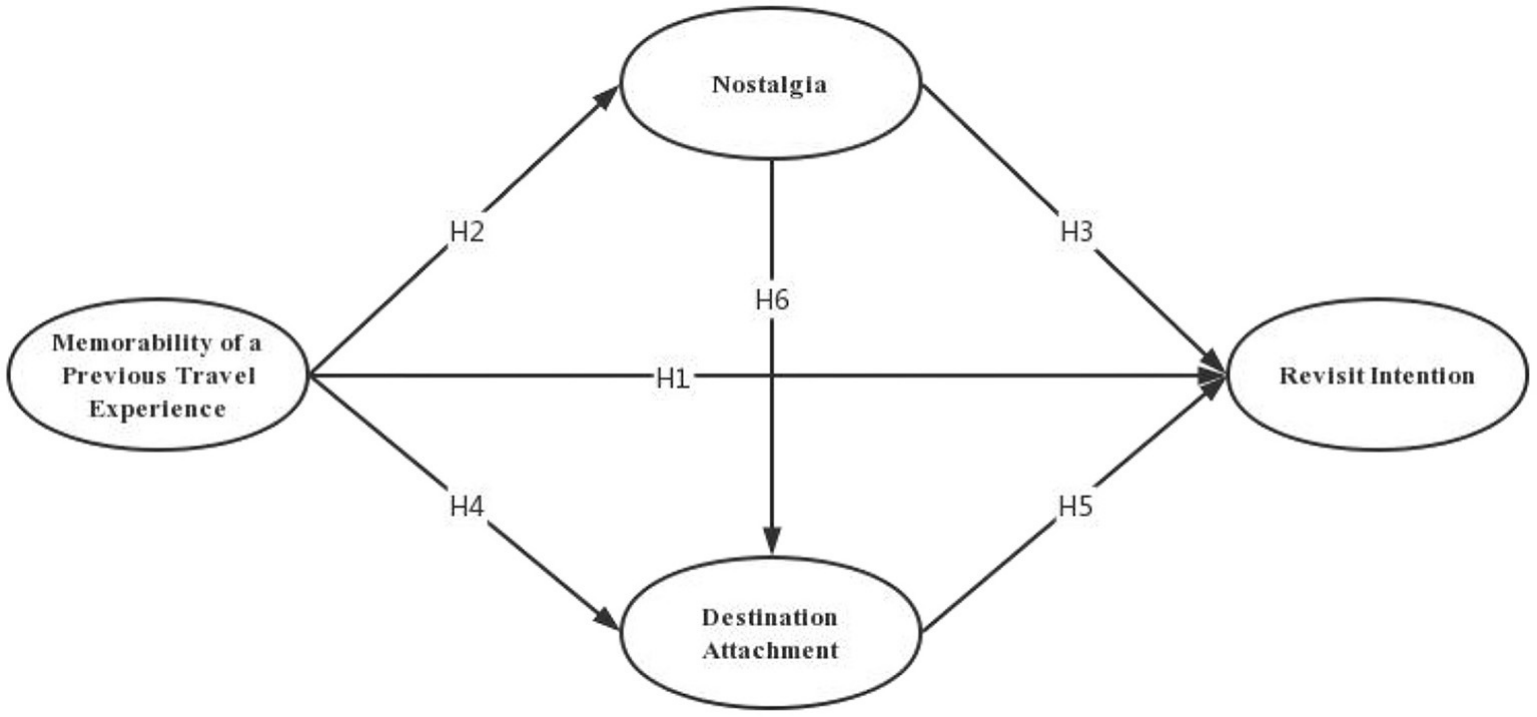
Research models.

The research model is shown in Figure 1.

## Methodology

### Research setting

Over the past two decades, Chinese outbound travel has grown phenomenally, becoming the world's largest outbound market (Ministry of Culture Tourism, PRC, 2019) China's outbound tourism business expanded to 149 million trips in 2018, with outbound travelers spending more than $130 billion overseas, according to the China Tourism Academy (China Tourism Academy, 2019), which shows huge potential China has as a tourist source country. And many countries are eager to create focused plans to draw the entrance of Chinese visitors (Huang and Wei, 2018). The COVID-19 pandemic has caused the global tourist sector to stall, and the travel preference of Chinese tourists has changed dramatically, that is, from overseas travel in pursuit of novel experiences to choosing familiar and safe domestic destinations as their first choice for travel (Sun et al., 2022). As Chinese tourists make up the largest part of the global outbound tourism industry, a strategy to bring them back to the destination will be crucial to the recovery of the local tourism industry.

As the fourth biggest bay area in the world, the Guangdong-Hong Kong-Macao Greater Bay Area is now one of China's most open and economically active areas. The Guangdong-Hong Kong-Macao Greater Bay Area's three cities with the best economic development are Guangzhou, Shenzhen, and Foshan. These cities contribute significantly to the nation's economy's fast growth, with city GDPs in excess of trillions of yuan. The tremendous expansion of outbound tourism is mostly a result of economic growth and rising national disposable income (Huang et al., 2015; Huang and Wei, 2018). Therefore, this study chooses Guangzhou, Shenzhen, and Foshan as the sample collection sites.

### Measurement scales and questionnaire design

The measurement scales that have been validated through empirical studies were borrowed to avoid measurement flaws caused by individual questions. The measurable items for nostalgia, revisit intention and MPTE were taken from Sthapit et al. (2022b), Bi et al. (2020) and Hu and Xu (2021), respectively. While destination attachment was adapted to meet the current research situation from Reitsamer et al. (2016). A total of 19 items (see Table 1) were evaluated using a 7-point Likert scale, with 1 indicating completely disagree and 7 indicating completely agree.

**Table 1:** Measurement scales.

	**Measured item**
	**Memorability of a previous travel experience (MPTE)**
MPTE1	The experience was special to me.
MPTE2	The experience was memorable to me.
MPTE3	The experience was favorable to me.
MPTE4	The experience was valuable to me.
MPTE5	The experience was meaningful to me.
MPTE6	The experience was important to me.
MPTE7	The experience was unique to me.
	**Nostalgia (NO)**
NO1	Thinking about this experience makes me feel nostalgic.
NO2	Thinking about this experience brings back my good memories.
NO3	Right now, I am feeling quite nostalgic.
NO4	Right now, I am immersed in fond memories.
	**Destination attachment (DA)**
DA1	That place is the best place for what I like to do on holidays.
DA2	I am very attached to that place.
DA3	Holidaying in that place means a lot to me.
DA4	No other place can provide the same holiday experience as that place.
	**Revisit intention (RI)**
RI1	I intend to revisit that place in the near future.
RI2	I plan to revisit that place in the near future.
RI3	I would like to visit that place in the near future.
RI4	I probably will revisit that place in the near future.

Three sections make up the research questionnaire. The screening question is in the first section, by answering “Have you ever traveled abroad? (Excluding Hong Kong, Macao, and Taiwan)” to determine the eligibility of the participants. Only individuals who replied “yes” to this inquiry will be given the opportunity to fill out the survey. Respondents were asked to recall their most recent memorable travel experience to an overseas destination before responding to the second part of the questionnaire, which was used to measure items in four constructs. The third section contained background information about the participants. This study eliminated translation bias by translating the English questionnaire into Chinese and then transcribing it into English. Then, two professors in tourism were invited to verify the content of both English and Chinese versions. A pre-test with 50 participants was conducted in Guangzhou on May 26, 2022, to further confirm the validity of the questionnaire content. All participants indicated that they were able to clearly understand the research questions after completing the questionnaire, and therefore, the questionnaire was not further modified.

### Data collection

From June 5 to June 11, 2022, six trained research assistants conducted daily questionnaire surveys in shopping malls in three Greater Bay Area cities, Guangzhou, Shenzhen, and Foshan. This study used systematic sampling to conduct the research. From 11:00 to 21:00, the study assistants took turns choosing one in 10 passersby to complete the questionnaire in the malls. The study assistants would wait for 10 more people if the respondents were not Chinese citizens or declined to respond for any other reason. A total of 387 samples were collected. Ninety-six questionnaires were removed due to incomplete responses from some participants and similar ratings on most items. The participant profiles are shown in Table 2. The majority of the respondents (62.2%) had a bachelor's or junior college degree and aged between 26 and 35 years (36.4%) and 18–25 years (29.2%), 63.2% were female and 36.8% were male. Meanwhile, most respondents said their monthly income is between 5,001–15,000 yuan (47.8 percent), which is similar to the per capita distributable income data published by Guangdong Province in 2021 (People's Government of Guangdong Province, 2022). In addition, over 40% of respondents travel overseas more frequently than once in 2018–2019 (2 years) (42.7%).

**Table 2:** Sample profile (*n* = 291).

		**Frequency**	**Percent**
Gender	Males	107	36.8
	Females	184	63.2
Age	18–25	85	29.2
	26–35	106	36.4
	36–45	39	13.4
	46–55	56	19.2
	56–65	5	1.7
Frequency of	Only travel before 2018	104	35.7
outbound travel	One	63	21.6
(2018–2019)	Two	70	24.1
	Three to four	16	5.5
	Over four	38	13.1
Personal monthly	≤ 5,000	69	23.7
income (RMB)	5,001–15,000	139	47.8
	15,001–35,000	57	19.6
	>35,000	26	8.9
Education	Below high school	10	3.4
background	High school or technical secondary school	26	8.9
	Bachelor or junior college degree	181	62.2
	Master degree or above	74	25.4

## Results

### Outer model analysis

For each measurable question item, factor loadings are included in Table 3 along with descriptive statistical analysis results. The minimum PLS factor loading value was 0.746 (>0.700). Cronbach's alpha and CR values of all variables are above 0.7, and AVE values are above the suggested threshold of 0.5, as shown in Table 4, which indicates that the structure has good validity and reliability (Hair et al., 2010). In addition, Table 4 shows the results of Fornell-Larcker Criterion and Heterotrait-Monotrait Ratio (HTMT) data, The square root of AVE for each construct in this study is greater than the correlation between paired correlations between constructs (Fornell and Larcker, 1981), and HTMT ratios were < 0.90 (Henseler et al., 2015), indicating that the discriminant validity was also satisfactory.

**Table 3:** Descriptive statistics and factor loadings.

	**Mean**	**S.D**.	**Kurtosis**	**Skewness**	**Loadings**
DA1	5.595	1.255	1.148	−1.034	0.870
DA2	5.876	1.080	0.866	−0.903	0.830
DA3	5.660	1.278	1.589	−1.149	0.832
DA4	5.430	1.341	0.875	−0.932	0.843
MPTE1	5.966	1.055	0.709	−0.903	0.862
MPTE2	6.062	1.010	0.948	−1.050	0.873
MPTE3	6.021	0.984	0.820	−0.889	0.849
MPTE4	5.907	1.030	0.625	−0.781	0.878
MPTE5	5.973	1.028	0.349	−0.840	0.889
MPTE6	5.759	1.197	0.972	−0.952	0.826
MPTE7	5.605	1.339	0.826	−1.012	0.746
NO1	5.948	0.985	0.807	−0.873	0.864
NO2	6.052	0.967	0.183	−0.814	0.892
NO3	5.811	1.202	1.225	−1.086	0.884
NO4	5.660	1.331	1.328	−1.134	0.853
RI1	6.117	1.094	1.982	−1.422	0.906
RI2	6.086	1.141	3.106	−1.592	0.880
RI3	6.186	1.094	3.849	−1.737	0.926
RI4	6.261	1.081	5.597	−2.126	0.920

**Table 4:** Reliability, construct validity, and correlation.

	**Cronbach's alpha**	**CR**	**AVE**	**Fornell-Larcker criterion**				**Heterotrait-Monotrait ratio**		
				**DA**	**MPTE**	**NO**	**RI**	**DA**	**MPTE**	**NO**
DA	0.811	0.888	0.725	0.844						
MPTE	0.940	0.957	0.847	0.572	0.847			0.635		
NO	0.812	0.876	0.639	0.604	0.767	0.873		0.680	0.838	
RI	0.897	0.936	0.830	0.498	0.556	0.552	0.908	0.542	0.590	0.600

AVE, average variance extracted; CR-construct reliability, Italic front-square-root of AVE.

### Inner model analysis

**Figure 2:**
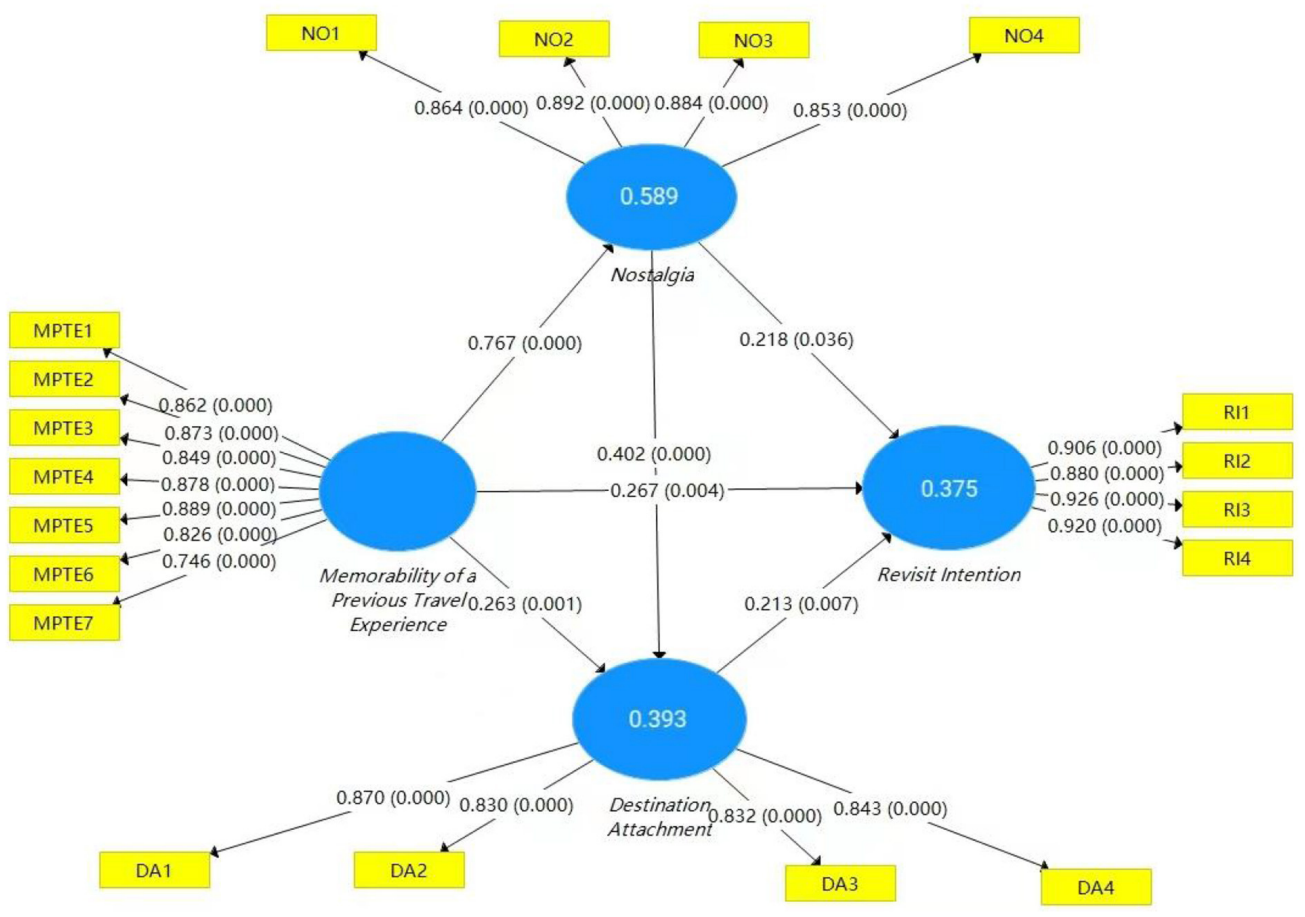
Results of PLS-SEM analysis.

Bootstrapping (5,000 Subsamples) was used to verify the hypothesis model. Figure 2 shows the results of the PLS-SEM analysis. There are two main reasons for using PLS-SEM in this study. On the one hand, compared with CB-SEM analysis, PLS-SEM has no mandatory requirement on the normal distribution of samples. On the other hand, the focus of this study is not on the comparison between theories, but on predictive research (Hair et al., 2017). Therefore, PLS-SEM was used to analyze the research model in this study. The results showed that MPTE, nostalgia (NO), and destination attachment (DA) had influence on revisit intention (RI) (β = 0.267, *p* = 0.004; β = 0.218, *p* = 0.036; β = 0.213, *p* = 0.007). MPTE had a significant effect on NO (β = 0.767, *p* < 0.001) and DA (β = 0.767, *p* = 0.001). NO had a significant influence on destination attachment DA (β = 0.402, *p* < 0.001). Meanwhile, R-squared values of NO, DA, and RI were 0.589, 0.393, and 0.375, respectively, which were all higher than 0.25. Therefore, H1, H2, H3, H4, H5, and H6 were all supported. In addition, the inner variance inflation factor (VIF) was used to check any multicollinearity problem. As shown in Table 5, all VIF values are < 3.3, so there was no collinearity issue (Kock and Lynn, 2012).

**Table 5:** Results of hypotheses testing.

	**Coefficient**	**T-statistics**	**f-Square**	**VIF**	**Test results**
H1 DA → RI	0.213	2.781	0.044	1.648	Supported
H2 MPTE → DA	0.263	3.199	0.047	2.433	Supported
H3 MPTE → NO	0.767	24.887	1.433	1.000	Supported
H4 MPTE → RI	0.267	2.850	0.045	2.547	Supported
H5 NO → DA	0.402	4.606	0.109	2.433	Supported
H6 NO → RI	0.218	2.173	0.028	2.698	Supported

### Mediating effect on nostalgia and destination attachment

In this study, PLS Bootstrapping (5,000 Subsamples) was used to verify the mediating effect of NO and DA. Table 6 shows that both NO and DA had a significant mediating effect on the relationship between MPTE and BI. Since NO also mediated the effect of MPTE on DA, therefore, NO and DA played an indirect chain effect between MPTE and RI.

**Table 6:** The mediation effect of place identity and life satisfaction.

	**Effect**	**T value**	**2.5%**	**97.5%**	**Mediation**
MPTE → NO → DA	0.308	4.587	0.182	0.437	Partial mediation
MPTE → DA → RI	0.056	2.103	0.013	0.115	Partial mediation
MPTE → NO → DA → RI	0.066	2.228	0.019	0.135	Partial mediation
NO → DA → RI	0.086	2.269	0.025	0.170	Partial mediation
MPTE → NO → RI	0.168	2.172	0.031	0.324	Partial mediation

## Discussion and conclusions

### Conclusions

This study aimed to examine the influence of MPTE on revisit intention, considering the mediating role of nostalgia and destination attachment. The results supported all the hypotheses. First, MPTE positively affects revisit intention. This result was consistent with the study by Cifci (2022) that stimulating tourists' unforgettable and meaningful travel memories have a positive effect on enhancing tourists' revisit intention. Second, nostalgia and destination attachment play a mediating role between MPTE and tourists' revisit intention. This implies that tourists can be impressed by the long-term travel experiences of a certain destination. On one hand, they have a feeling of nostalgia resulting from repeating those long-term memories. On the other hand, they have a feeling of attachment to that destination. Finally, the strong nostalgia and destination attachment stimulates revisit intention among tourists. These results supported Sthapit et al.'s (2022b) findings that tourists' revisit intention becomes stronger with the strengthening of destination attachment. Third, nostalgia has a positive effect on destination attachment. This result is consistent with Io and Wan (2018) study that nostalgia is positively related to emotional destination attachment. Therefore, nostalgia, a memory longing for the past, leads to an affective sequence of emotional connections between a tourist and a tourist destination.

### Contributions

First, previous studies mainly examined the effect of short-term or temporary memorable travel experiences just after completing the trip (e.g., Sthapit et al., 2020). This study contributes to memorable travel experience research in examining the effect of long-term memorable travel experiences on tourists' travel decision-making. This stud provided evidence that tourists even left overseas destinations over 3 years, their long-term memory of travel information stored can still have the function of predicting their future travel behaviors. This study stimulated researchers' interest in studying the impact of long-term memory travel experiences on tourists' attitudes and behaviors.

Second, although researchers have explained the importance of welcome back tourism for the tourism recovery from the COVID-19 pandemic (Eran and Eli, 2021), we know little about how it works. This study contributes to welcome back tourism research by providing a research framework that clarifies the mechanism of how past travel experiences transformed into future revisit intentions. This study supported CAT that cognitive appraisals of experiences trigger emotions that influence behavioral responses. This study indicated that unforgettable travel experiences do not fade over time and effectively provoke positive emotions under certain circumstances, including triggering nostalgia and reinforcing the emotional connection of attachment between tourists and the destination, which leads to tourists' revisit intentions. This study provided researchers a research framework that can be extended for further studying welcome back tourism.

Third, some studies on nostalgia considered past nostalgia as a negative emotion (Sedikides and Wildschut, 2016) but some treated it as a positive emotion (Sedikides et al., 2015). This study confirmed that nostalgia is an individual's positive emotion stemming from a positive travel experience in tourism research. This study further confirmed that emotion stemming from past memory (nostalgia) has a facilitative effect on enhancing the positive emotional connection (destination attachment) that individual forms with a particular destination. Although there are some recent studies on nostalgia in a tourism research context (e.g., Hu and Xu, 2021), we do not know much about the effect of nostalgia on tourist attitudes and behaviors. This study indicated that nostalgia and destination attachment act as chain mediators between cognitive travel experiences and behavioral intentions. This study contributes to our knowledge of how past nostalgia works with destination attachment in motivating tourist behavioral intentions. It provides researchers with the literature regarding nostalgia in tourism research.

### Recommendations

The results of this study have important practical implications for the marketing strategies of tourist destinations after the COVID-19 pandemic. First, destination marketers can add nostalgic elements in advertisements to evoke tourists' nostalgic feelings. For example, presenting the special meaning of a destination through short videos of various narrative themes that tourists had experienced. It releases a welcome back signal and strengthens tourists' emotional connection.

Second, in the future, destination managers should focus on making tourists' travel experiences inspiring and sustainable. For enhancing the profundity of tourists' experiences, providing more participatory and creative tourism activities can create more memorable experiences for tourists. For example, a parade with local culture. Participatory and creative tourism activities can make tourists' memories last longer.

Finally, for the post-COVID-19 tourism recovery, destination managers should develop a welcome back marketing strategy to restore the destination to its former vitality by attracting repeat visitors. The promotion strategy includes a special discount on tourism products such as hotel accommodations and air tickets for repeated tourists. The welcome back strategy should focus on tourists' memories of previous travel experiences and strengthen their nostalgia and destination attachment emotions. In addition, the welcome back strategy should include attracting other repeat tourists through online user-generated content (UGC) on social media from existing repeat tourists during travel, allowing UGC to evoke the memories of other repeat tourists.

### Research limitations and future research

Although the present study developed a theoretical model to examine the relationship between MPTE, nostalgia, destination attachment, and revisit intention to understand causality, more research is needed in the future to further validate as well as extend these concepts. First, this study used a single dimension to measure the MPTE. Future research could measure the MPTE in multiple dimensions, such as engagement, novelty, meaningfulness, etc. (Kim et al., 2012; Tsai, 2016). Second, the sample of this study is limited as it was only for Chinese tourists. Future studies can sample countries with different cultural backgrounds to verify the generalizability of the model. Finally, this study only constructed a basic model based on travel memories, positive emotions, and revisit intention, on which future studies can be expanded, for example, by considering whether there are potential moderating influences.

## Data availability statement

The original contributions presented in the study are included in the article/supplementary material, further inquiries can be directed to the corresponding author.

## Ethics statement

Ethical review and approval was not required for the study on human participants in accordance with the local legislation and institutional requirements. Written informed consent from the participants to participate in this study was not required in accordance with the national legislation and the institutional requirements.

## Author contributions

YL contributed to the development of the research framework and the supervision of research. IKWL directed the writing of the manuscript and performed data analysis. XYL is responsible for collecting data. XYL and YL drafting the manuscript. YL, IKWL, XW, and XYL revising the manuscript. XW is responsible for managing manuscript submission. All authors contributed to the article and approved the submitted version.

## Conflict of interest

The authors declare that the research was conducted in the absence of any commercial or financial relationships that could be construed as a potential conflict of interest.

## Publisher's note

All claims expressed in this article are solely those of the authors and do not necessarily represent those of their affiliated organizations, or those of the publisher, the editors and the reviewers. Any product that may be evaluated in this article, or claim that may be made by its manufacturer, is not guaranteed or endorsed by the publisher.
